# Emotions in play: young people’s and clinicians’ experience of ‘Thinking about Emotions’ group

**DOI:** 10.1007/s40519-019-00646-3

**Published:** 2019-02-08

**Authors:** Lucia Giombini, Sophie Nesbitt, Jenni Leppanen, Hannah Cox, Anna Foxall, Abigail Easter, Kate Tchanturia

**Affiliations:** 1 Rhodes Wood Hospital, Elysium Healthcare, Sheperd’s way, Brookmans Park, Hatfield, London, AL96NN UK; 2 Department of Psychological Medicine, Institute of Psychiatry, Psychology and Neuroscience, King’s College London, London, SE58AF UK; 3 Research and Population Research Department, Institute of Psychiatry, Psychology and Neuroscience Health Services, PO59 King’s College London, London, UK; 4 Department of Psychology, Illia University, Tbilisi, Georgia; 5 South London and Maudsley NHS Foundation Trust, London, UK

**Keywords:** Anorexia nervosa, Emotion skills training, Young people, Clinicians’ experience, Service users’ experience

## Abstract

**Purpose:**

Emotional difficulties in young people (YP) with anorexia nervosa (AN) are well recognised. Improved strategies are needed to support inpatients to tolerate group therapy and to help them to better identify and manage their emotions. Cognitive Remediation and Emotion Skills Training (CREST) for AN adults, aimed at improving emotional processing skills, has been found beneficial in adult AN groups. A case series of CREST was conducted in an inpatient ward for YP (CREST-YP) to evaluate its suitability for a younger population.

**Methods:**

A mixed-methods assessment was used. Thirty-two YP and 3 facilitators took part in qualitative interviews. YP (*n* = 32) also completed pre- and post-self-report questionnaires assessing emotional functioning.

**Results:**

Preliminary qualitative results showed that YP found it helpful to learn about emotion processes. More support is needed to clarify the link between emotions and AN. Quantitative results showed no significant changes in YP’s self-perceived emotional functioning. Although no statistically significant changes were observed, a small increase in YP’s use of both reappraisal (standardised mean changes scores, SMCC 0.22) and suppression (SMCC − 0.22) as a means to regulate their emotions was found.

**Conclusions:**

Pilot findings suggest that CREST-YP is a suitable intervention for YP with AN. Age-appropriate adaptations are needed to improve YP’s engagement in group CREST.

**Level of evidence:**

Level IV: Evidence obtained from multiple time series.

## Introduction

Emotional regulation is significantly impaired among individuals with anorexia nervosa (AN), and has been shown to be a key contributing factor to the maintenance of the illness. Patients with AN have difficulties with intimacy, attachment, self-referential processing and body perception [1]. They experience emotions and social interactions as highly problematic, showing high rates of alexithymia [2, 3] and emotional avoidance, and poor facial expressivity, which can perpetuate AN symptomatology [4, 5]. Poor awareness of emotions, as well as cognitive inflexibility, may be an obstacle to recovery in AN [6, 7].

Identifying effective interventions that target emotional processing is therefore vital to improving long-term outcomes in this group.

Interventions targeting emotional processing have been explored in AN populations, mainly in adults and adolescents. Within the Dialectical Behaviour Therapy (DBT) framework, Radical Open DBT (RO-DBT) has been specifically applied to AN populations, targeting openness, flexibility and social connectedness. RO-DBT, consisting of both individual and group skill sessions, comprises 30 lessons [7]. However, with a drive towards shortening the length of inpatient treatment, in line with the recent NICE guidelines [8], most therapeutic programmes would find it difficult to accommodate longer interventions.

Emotion Acceptance Behaviour Therapy (EABT) is described as a novel psychotherapeutic intervention for older adolescents and adults with AN. The intervention is designed to increase emotion awareness and decrease emotion avoidance. However, it is aimed to be delivered individually within outpatient settings over a range of 40–50 weeks [4].

Cognitive Remediation and Emotion Skills Training (CREST) was developed with the purpose of addressing emotion processing and social skills [3]. It was conducted in individual (eight sessions) and group (five sessions) formats in adults with AN in inpatient and day care treatment settings. CREST, in an individual format, addresses difficulties with cognitive styles and emotional awareness, recognition and expression. It is particularly suitable in inpatient settings where patients might have a limited motivation to change symptomatic eating-related behaviours, a limited ability to use complex therapies and a limited amount of time due to short hospital admissions. CREST in a group format consists of five sessions and focuses on psychoeducation and simple experiential exercises to allow patients to practice some skills in a social group environment. In adult populations, it has shown promise in case series to significantly improve patients’ self-reported social anhedonia and has received positive qualitative feedback from both patients and clinicians [3]. Similar improvements in social anhedonia and in alexithymia have been reported following CREST in an individual format with small effect sizes [9].

Preliminary research suggests that CREST might be a valuable brief intervention to help improve patients’ engagement in treatment and awareness of their emotions and provide them with a helpful toolbox to regulate emotions and to engage in other therapies [10]. Research has also indicated that CREST is suitable for delivery within inpatient and outpatient settings; however, current evidence is limited to adult populations and case series [11].

The present study aims to assess the suitability of CREST-YP (CREST for young people) in a group format in a child and adolescent eating disorder (ED) inpatient unit, by conducting a mixed-method assessment. It explores the experience of young people (YP) with AN in receiving CREST-YP, enriched by the point of view of the facilitators. Furthermore, the study aims to evaluate YP’s emotional functioning pre and post the intervention. This study will inform the direction for the development of the intervention with age-appropriate materials for YP.

## Methods

### Setting and participants

The study was conducted in a specialist inpatient ED service for YP aged 6–18 years requiring hospital treatment for ED. Patients are admitted to the hospital when they can no longer safely remain in the community. The service offers a multi-disciplinary treatment including individual therapy, family therapy and group therapy.

In 2017, group CREST-YP was included as part of the treatment programme. Inclusion criteria involved all YP admitted to the clinic between January 2017 and May 2017, with a diagnosis of AN, under their and their families’ informed consent. CREST-YP was offered in conjunction with the multidisciplinary treatment indicated above.

A total of 32 participants aged 11–17 years engaged in CREST-YP. All had a diagnosis of AN based on DSM-5 criteria, assessed by the consultant psychiatrist at the admission [12].

All participants received individual cognitive behaviour therapy and family therapy using a systemic model. Participants received the following group therapies: 84.38% (*N* = 27) anxiety management, 96.88% (*N* = 31) relaxation, 90.63% (*N* = 29) adapted motivation enhancement, 81.25% (*N* = 26) relapse prevention, 28.13% (*N* = 9) food psychoeducation and 34.38% (*N* = 11) recovery management. Additionally, some of the participants received pharmacotherapy: 12.51% (*N* = 4) were prescribed antidepressant, 18.75% (*N* = 6) were taking antipsychotics, 9.38% (*N* = 3) were taking both an antipsychotic and an antidepressant. Young people prescribed supplements for vitamin deficiencies were 34.38% (*N* = 11) and sleeping medications 12.5% (*N* = 4).

The weight for height (WfH) percentage was measured at the admission and reviewed weekly. All the participants in this study gained weight via eating or nasogastric feeding conducted under the Mental Health Act (2007) at a consistent rate of between 0.8 and 1 kg per week. For this reason WfH was not included in the analysis.

All YP completed the group cycle (no attrition).

### Procedure

#### The intervention

CREST-YP was offered at the beginning of the treatment programme. The structure of the group was based on the manual ‘Cognitive Remediation and Emotional Skills Training—Inpatient pack-Part II’ developed by Tchanturia and colleagues [9]. The group consisted of five weekly sessions, covering the following themes: (1) recognising positive emotions; (2) the nature and function of emotions; (3) how do we identify emotions; (4) emotion expression vs. emotion suppression; (5) emotions and needs. Each session lasted 1 h with a balance between discussion, experiential exercises and psycho-educational materials. A psychotherapist and two assistant psychologists delivered and facilitated the sessions. This work was supervised weekly by a clinical psychologist. To the authors’ knowledge, there was no other manual for YP with AN that focuses on emotion processing, which is applicable in an inpatient setting; therefore, the CREST manual developed for adults with AN was used.

### Measures

#### Semi-structured interview

YP met the interviewer (AF) for 45 min at the end of the intervention and they were asked to share their thoughts and feedback about the group. YP were asked to elaborate their views regarding: what they found beneficial and challenging; what they enjoyed and what they did not like; whether the group helped them to manage their ED; and suggestions for further improvements of the intervention. The interviews were conducted in a therapy room in the ward, audio-recorded and subsequently transcribed. Furthermore, three facilitators were interviewed by the fifth author and they were asked to provide their views on: their experience of facilitating the group; YP’s experiences of the group; homework assignments; the impact of the group on the ED; and suggestions for improvement. The interview was created ad hoc, as an initial exploratory approach was required for the first time evaluation of CREST-YP. The interviews were devised by the research and clinical psychology team. They used open-ended questions to encourage detailed descriptions of participants’ experience. We also used interview items from evaluation of CREST in the adult population [3, 10]. The interview is outlined in “Appendix 1”.

#### Satisfaction questionnaire (SQ)

The SQ is a three-item self-report five-point Likert scale (0 = not at all; 5 = very much), which was used to evaluate the extent to which YP found the sessions enjoyable, useful and educational. SQ was administered at the end of the CREST cycle. The questionnaire was devised for the purpose of the study based on both the hospital’s protocol for evaluating group therapies and satisfaction questionnaires used in previous studies evaluating CREST in the adult population [3, 10]. The satisfaction questionnaire is outlined in “Appendix 2”.

#### Emotional Regulation Questionnaire for Children and Adolescents (ERQ-CA)

The ERQ-CA [13] is a ten-item self-report Likert scale, which was used to measure YP’s tendency to regulate their emotions through cognitive reappraisal (four items) and expressive suppression (six items).

#### Revised Social Anhedonia Scale (RSAS)

The RSAS [14] with 40 self-report items was administered to assess the social dimension of anhedonia.

#### Toronto Alexithymia Scale (TAS-20)

The TAS-20 [15] is a 20-item self-report measure which was used to evaluate difficulties YP may have with identifying feelings (7 items), describing feelings (5 items) and externally orientated thinking (8 items).

#### Motivation Ruler (MR)

The MR [16] measured YP’s motivational readiness to change through two ten-point Likert scales assessing self-reported importance of change (MR-I) and ability to change (MR-A).

### Data collection

At the beginning and end of the cycle, YP were asked to complete a series of outcome measures to assess aspects of their self-perceived emotional functioning and their overall satisfaction with the group. Additionally, YP were asked to take part in a semi-structured interview.

### Data analysis

#### Thematic analysis

YP’s and clinicians’ interviews conducted at the end of the group were analysed using thematic analysis, a widely used qualitative analytic method [17]. All interviews were transcribed by members of the psychology team not directly involved with delivering the intervention. They were anonymised and given an identifiable code. Interviews were read repeatedly by both authors so that they could familiarize with the general content. Initially, the first and fifth author analysed separately the semantic level of the data. To provide a less biased interpretation, the fifth author examined the interviews without knowledge of the YP and their engagement.

They familiarised themselves with the data generating initial codes, then organised the data to show relevant themes. In the subsequent phase, an understanding of the themes was sought. Key words were identified for each interview and listed separately. Key words were then organised in higher-order themes, each with lower-order themes. The first and fifth author met to discuss and analyse these. Both authors felt that data saturation had been reached and no new themes were identified in the data.

#### Quantitative analysis

All statistical analysis was conducted with R [18]. Linear mixed effects analysis from the package lme4 [19] was used to examine changes in MR-I, MR-A, RSAS, TAS and ERQ-CA following the intervention. Age was added to all linear mixed effects models as a covariate to control for differences in maturity and brain development. Effect sizes were estimated by calculating standardised mean changes scores (SMCC).

## Results

### Sample characteristics

The final YP sample consisted of 32 participants (28 females, 4 males); the mean age was 14.03 years (SD 1.75, range 11–17). The weight for height (WfH) percentage was measured at the beginning of CREST-YP (mean 75.68; SD 6.44, range 61.53–85.92). Of participants, 26 (81.25%) were white British; 3 (9.38%) white European; 2 (6.25%) other mixed; and 1 (3.13%) Caribbean. They were all enrolled in secondary school. Comorbidities present in the sample included: generalised anxiety disorder 12.5% (*N* = 4), autism 3% (*N* = 1), social anxiety disorder 3% (*N* = 1), separation anxiety 3% (*N* = 1) and 6% (*N* = 2) had experienced depressive episodes. Five participants had one previous admission to a paediatric unit and one participant had two admissions to paediatric units prior to their inpatient admission.

### Qualitative analysis results

Higher-order themes and sub-themes for both YP and facilitators were identified. Results are reported in Tables 1 and 2. Pseudonyms are used to maintain YP anonymity. Due to the small size of the facilitators’ sample (*N* = 3) and the consistency of their feedback, frequencies are not reported.

**Table 1:** Young people experience of CREST-YP: higher-order themes and lower-order themes

Higher-order themes	*N* (%) YP	Lower-order themes	*N* (%) YP
Exploring emotions	32 (100)	Importance of identifying and understanding emotions	14 (43.75)
		Difficulty in sharing emotional experiences	18 (56.25)
Emotions and eating disorder	18 (56.25)	Group felt unrelated to eating disorders	11 (34.37)
		Helpful in managing emotions related to eating disorder	7 (21.87)
Homework	21 (65.62)	Unnecessary in completing homework	15 (46.87)
		Tool for reinforcing learning	6 (18.75)
Suggestions for improvement	31 (96.87)	Need for visual tool and interactive activities in smaller groups	18 (56.25)
		Need for diverse content	13 (40.62)

**Table 2:** Facilitators’ experience of CREST-YP: higher-order themes and lower-order themes

Higher-order themes	Lower-order themes	Quotes
Helpfulness	Differentiating thoughts and feelings	I think the YP liked the fact that some information and knowledge they might not have heard of before, meaning they actually managed to learn something new and useful from the group. An example of this might be a certain coping strategy in managing a particular emotion
	Learning about coping strategies	
	Learning about functions and effects of emotions	
Relevance of emotions	Value of emotions in everyone’s life	The topics of the sessions felt very relevant given that emotions are such a big part of our everyday life. Therefore you’re able to share personal experiences of a given emotion. So, I feel perhaps better able to understand and relate to the contents of the CREST group and I wonder if the young people see that too, that you are on the same page and that as facilitators we experience these emotions too
	Increased connection between facilitators and YP with regard to emotions	
	Less challenging talking about emotions compared to ED	
The link with the ED	Difficult to assess	A few of the older children, aged 15 and above, shared that they felt the group had impacted upon their ED and were able to make the link between the ED and emotions. However, with the younger ones, just from looking at the feedback we get each week, I don’t think this is the case
	Increased willingness to ask for support	
	Helpfulness in managing the anxiety at the core of the ED	
The experience of homework	Easy to forget	Some of the YP did do the homework, it tended to be the same YP each week. In general the majority of the YP did not do the homework each week. I do think that not doing it was often more to do with forgetting though rather than due to the content. Often the response of YP was quite dismissive to homework being set
	Only few YP showed commitment	
	Signals of boredom and annoyance	
Specifics of children vs. adolescents	Difficulties in understanding the link between emotions and ED	The sessions are very discussion based. This works better with the older kids and when the group are engaging and verbally contributing. It doesn’t work so well with the younger ones who don’t feel as comfortable talking in front of the group
	Preferences for interactive games and videos	
	Preferences for small group work	
	Less verbal interactions	
Suggestions for further improvements	Discussions and activities in smaller groups	I think definitely to look at the use of child friendly cognitive games. Maybe also less information in some of the sessions so there is more time to split into smaller groups and feedback as I think smaller group work improves overall engagement with the session
	Increased number of games and use of videos	
	Increased child-friendly material	
	Increased exploration of positive emotions	
	Make the link between emotions and ED clear	

### Young people experience

Overall, four common themes were reported by YP.

#### Exploring emotions

All YP commented on the importance of talking about emotions. Fourteen YP (43%) emphasised the value of learning how to better identify emotions. For example, Lucy (17 years old) said: “I think it made me think more about the way I act when I’m in a stressful situation, or angry, or in anxiety and realise that there are ways to cope and to manage those feelings” Frances (16 years old) added: “the group made me understand the importance of being more open about your emotions and not hiding them”. Eighteen YP (56%) shared their difficulties regarding talking about emotions with the others.

#### Emotions and ED

Eleven YP (34%) found that the group was not directly addressing the ED symptoms. Seven YP (21%) specified that the group helped them to understand the emotions related to the ED symptoms, but it was not helpful to better cope with it. For example, Laura (17 years old) reported: “I feel they have helped me understand the emotions linked with my disorder, but not helped me with it entirely”.

#### Homework

At the end of each session, participants were assigned homework; however, the YP often did not do it. Fifteen of them (46%) perceived it as not necessary (Amy, 14 years old: “I never did any. It just didn’t really seem that necessary to me. If it was fun then I guess I would take a look at it.”), while 6 YP (18%) said that it was a good tool to reinforce learning.

#### Suggestions for improvement

Thirty-one YP (96%) were able to provide some suggestions for further developments. Eighteen YP (56%) recommended the use of visual tools and more experiential activities to be conducted in smaller groups. Lara, 12 years old, reported: “It would be helpful if the sessions could be more interactive, using more child-friendly material and practical tasks”. Thirteen YP (40%) suggested that the contents of the sessions could be more varied and less repetitive. Specifically, they would like to talk about positive emotions and learn more coping strategies.

### Facilitators’ experience

Six higher-order themes were identified for the facilitators’ experience of the group. Lower-order themes and quotes are reported in Table 2.

#### Helpfulness

Facilitators observed that YP were willing to learn how to identify different emotions and new coping strategies to help manage them.

#### Relevance of emotions

They perceived that there was a shared experience regarding the importance of emotions in everyday life between them and YP, who appeared to have felt being better understood.

#### The link with the ED

It was observed that YP were more willing to ask for support and to talk about their emotions with the nursing team when they were in the ward; however during the group, YP did not talk about the ED symptoms.

#### The experience of homework

Facilitators noticed that YP were willing to practice some homework between the sessions when it was not related to their emotions, whilst when the task was focused on emotions, they required more support.

#### Specifics of children vs. adolescents

It was observed that adolescents were more willing to participate to the discussion and reflect on their learning, while children seemed to better engage in interactive and experiential activities.

#### Suggestions for improvements

As YP, also facilitators suggested that it would be important to add further child-friendly interactive activities and facilitate the discussion in smaller groups of three people, as this would allow them to share more personal experiences. Also, it seemed important to help YP to make clearer links between emotions and ED symptoms.

### Quantitative analysis results

Changes in clinical outcome measures are presented in Table 3.

**Table 3:** Descriptive and statistical differences between Emotional Regulation Questionnaire for Children and Adolescents (ERQCA), Revised Social Anhedonia Scale (RSAS), Toronto Alexithymia Scale (TAS-20) and Motivational Ruler (MR) subscales pre- and post-CREST-YP

	Mean	SD	*F* statistic, *p* value	SMCC
ERQCA				
Reappraisal (pre)	22.28	7.3	Time: *F*(31) = 1.54, *p* = 0.225 Age: *F*(30) = 0.01, *p* = 0.907	0.22
Reappraisal (post)	23.94	6.98		
Suppression (pre)	15.34	3.98	Time: *F*(31) = 1.49, *p* = 0.231 Age: *F*(30) = 3.10, *p* = 0.088	− 0.22
Suppression (post)	16.22	4.22		
RSAS				
RSAS (pre)	13.94	8.52	Time: *F*(30) = 0.11, *p* = 0.748 Age: *F*(29) = 0.97, *p* = 0.332	0.06
RSAS (post)	13.62	8.82		
TAS-20				
Global score (pre)	58.44	11.70	Time: *F*(31) = 0.29, *p* = 0.593 Age: *F*(30) = 0.88, *p* = 0.354	0.10
Global score (post)	57.66	12.53		
MRI				
Importance to change (pre)	6.78	2.51	Time: *F*(31) = 0.01, *p* = 0.914 Age: *F*(30) = 1.16, *p* = 0.290	0.02
Importance to change (post)	6.81	2.61		
Ability to change (pre)	5.88	2.85	Time: *F*(31) = 1.22, *p* = 0.279 Age: *F*(30) = 0.12, *p* = 0.734	0.19
Ability to change (post)	6.20	2.70		

The results showed no statistically significant changes in YP’s self-reported emotional functioning, social anhedonia, alexithymia and motivation pre and post the CREST cycle (Table 3). Although no statistically significant changes were observed, a small increase in YP’s use of both reappraisal and suppression as a means to regulate their emotions was found. Post-CREST, the results additionally showed a negligible increase in YP’s perceived ability to change.

Sixty-two percent of YP reported enjoying the sessions, 56% of YP reported having found the sessions useful and 62% having used the strategies/skills learnt in the sessions.

## Discussion

Emotional regulation has been recognised to play a key role in YP’s ability to manage the developmental challenges they face. Both over- and under-regulation of emotions are assumed to imply risks for YP’s socioemotional adaptation, that is, for their ability to adjust to the socio-emotional challenges they encounter. Emotion regulation has been implicated in the development of diverse problems, including anxiety, depressive or conduct disorders and ED in YP [20]. One of the most significant maintaining factors in AN is a difficulty with ‘hot’ cognitions with high levels of social anhedonia and alexithymia poor facial expressivity [3, 5, 21, 22].

The aim of this case series study was to evaluate the suitability of a brief skills-based short intervention focused on emotion identification, expression and regulation for YP with AN. Amongst other interventions targeting emotion processing [4, 6, 7, 23], CREST seems to be applicable in ED inpatient settings.

This study is the first to explore the suitability of delivering a CREST group in YP with AN.

An overall positive qualitative feedback from both YP and facilitators was received. YP recognised that the group helped them to better identify their emotions and understand their functions. The facilitators also mentioned that YP were willing to learn new coping strategies to deal with emotions. This is consistent with what has been found in previous qualitative research [3, 10].

Some YP acknowledged that improved understanding and management of their emotions would lead them to better manage their ED symptoms. Facilitators observed that YP were more inclined to ask for support from the nursing team in the ward when struggling with ED worries, although they did not directly talk about the ED symptoms during the sessions. This feedback suggests that YP-CREST appears to have the potential to increase emotional awareness, openness and confidence, thus counteracting the emotional loneliness often experienced in AN [7].

Both YP and facilitators suggested that the addition of further interactive, experiential activities would encourage a more fluid group interaction, discussion and an overall better engagement. This need was also recognised within the adult population [2]. The engaging and collaborative style adopted by the facilitators in the current study was acknowledged by participants. Research conducted on the topic demonstrated that therapists’ friendliness and permissiveness have been proven to have a calming effect on patients’, as investigated by measuring physiological changes [24].

The quantitative results only partially supported the qualitative data. No statistically significant change was showed in YP’s self-perceived emotional functioning, social anhedonia, alexithymia and motivation to change. Small increases in YP’s use of both reappraisal and suppression subscales was noted as a means to regulate their emotions. The scores on the ERQ-CA, RSAS and the TAS-20 were roughly similar to those from other studies utilising a comparable sample of YP [e.g. 25, 26]. These findings are only partially consistent with the adult studies, where significant changes were found in social anhedonia [3] and motivation to change [11] after the CREST group.

Based on these preliminary results, CREST-YP could be further developed by presenting the topics described in the CREST manual [9] with the addition of visual and child-friendly tools and more experiential activities. Discussions and experiential tasks would benefit groups of three or four YP. The YP would also benefit from further information about the link between emotions and ED symptoms.

There are limitations in this study that should be addressed in future research. The CREST manual was originally developed for an adult population, and findings from this study suggest that further adaptation of YP is required with age-appropriate materials and interactive activities. Similarly, the RSAS and TAS-20 were validated using adult populations; however, they were administered to the YP for consistency with previous studies. Age-appropriate emotion processing assessments will be desirable in future studies.

A paradigm shift is occurring away from traditional talking therapies towards a range of novel targeted treatments, thus a transformation of the treatment landscape is taking place. A group of highly targeted treatments, based on neurobiological data/mechanisms (e.g. cognitive remediation treatment, exposure and neuromdoulation treatment) are now emerging [27, 28]. However, further research investigating the impact of specifically targeted interventions on emotion regulation is needed to corroborate these findings. From the current study, it seems that for the AN inpatient population, hot cognition is a challenging feature to address and modify psychological treatment [3]. Emotion regulation difficulties in AN do not seem to improve with weight restoration [29]. Further studies on YP with AN utilising fMRI to delineate the neural underpinnings of emotion recognition are needed to confirm whether the effects of starvation at a critical period of brain maturation could interfere with the normal developmental trajectory of emotion processing [30].

This study, as highlighted in previous research [31], confirms the necessity of using mixed-method approaches, which can consider not only the quantitative impact of an intervention on the YP’s emotional processing, but also the qualitative account of their experiences, to shape and improve the quality of interventions delivered. The use of patient feedback in developing services is considered a crucial aspect of clinical practice; it improves patient satisfaction and enables the development of a more effective, targeted inpatient group treatment programme for ED [32].
